# Editorial: Update in microcirculation in dermatology

**DOI:** 10.3389/fmed.2023.1285005

**Published:** 2023-09-21

**Authors:** Giusto Trevisan, Salvino Bilancini

**Affiliations:** ^1^Department of Medical Sciences, University of Trieste, Trieste, Italy; ^2^Research Center of Vascular Disease, Jean François Merlen, Frosinone, Italy

**Keywords:** Nailfold Capillaroscopy, Raynaud's, microcirculation, microangiopathies, connective tissue disease

The skin allows for the study of cutaneous and systemic diseases involving microcirculation. To this end, capillaroscopy, Laser-Doppler, and pCO_2_ are used.

In 1961, Marcello Malpighi observed small vessels in a frog, which joined the arterial and venous sides. Johan Christophorous Kolhaus used a primitive microscope to observe the small blood vessels surrounding the nails in 1663. Subsequently, in 1668, Herman Boerhaave analyzed the bulbar conjunctiva with a microscope. In 1862, Maurice Raynaud performed a nail capillaroscopy to evaluate the excessive vasospasm due to a physiological stimulus that resulted in ischemia of the fingers (1). Jean François Merlen (1912–1986) is considered the father of modern microcirculation (2).

Of these techniques, Nailfold Capillaroscopy (NC) is the most widely used, as the nailfold capillaries are arranged horizontally to the plane, while in the other areas of the epidermal surface, they are generally perpendicular. It is also a simple, non-invasive, repeatable, low-cost technique that allows to make measurements. The old Leitz Capillaroscope was already equipped with a reticle that permitted measurements. Video capillaroscopy currently allows precise measurements, including the diameter of the capillary branches (arteriolar, venular, and capillary top) (3). The capillaroscopic exam should be done after a 15-min stay in a room at 19–23°C. This technique is used in the diagnosis of connective tissue disease, Raynaud's phenomenon, vasculitis, Adamantiades-Behçet disease (Bergamo), diabetic microangiopathy, and red fingers' syndrome (4) but also in other conditions such as venous insufficiency and venous ulcers (Carpentier et al.) and psoriasis (5), in which the capillaroscopic examination is carried out above all the levels of the skin lesions under investigation (skin districts where the capillaries are vertical).

Capillaroscopy also has a specific pattern in some rare pathologies, such as Bürger's disease (Figure 1) and Rendu-Osler disease (examination of cutaneous telangiectasia).

**Figure 1:**
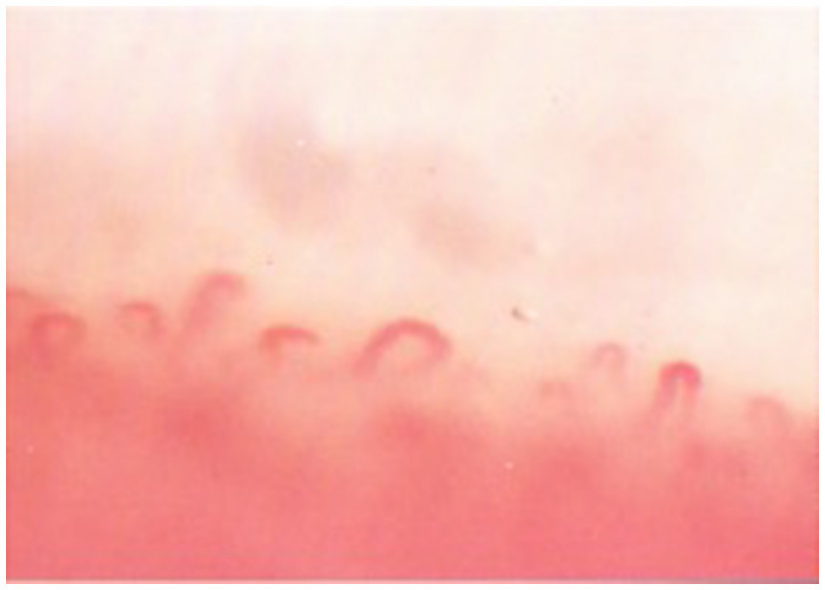
NF capillaroscopic pattern in the Burger's disease. The top of the capillary loops with an arched shape, with limited visibility of the arteriolar, and venular branches.

The NC allows a morphological and dynamic study to analyze the anomalies of the capillaries, the pericapillary spaces, the blood flow, and the reactivity of the capillaries (cold pressure test and hot test). The following features should be evaluated:

Quantitative: number of loops/mm [mean capillary density, indicative of microangiopathy if <8/mm; if <3/mm, it identifies desert areas (scleroderma)].

Qualitative:

∘ Minor dystrophic capillary loops are crisscross capillaries with at least two crossings, tortuous, notched, and ramified capillaries (type 5, 6, 7, 8);
∘ Capillary width: the range of the capillary dilatation is included between 30 and 50 μm;
∘ Major dystrophies: giant capillaries (capillary diameter >50μm) and regressing loops (scleroderma^8^ and dermatomyositis);
∘ Filiform loops (Raynaud's, anemia, hypotension);
∘ Telangiectasias [giant capillaries in scleroderma (Wang et al.) and dermatomyositis, vertically arranged are observed in Rendu-Osler disease];
∘ Capillary length: normally included between 250μm and 700μm; loops >700μm can be observed in SLE;
∘ Parallelism of the capillary loops: It can be lost in some connective tissue diseases, such as SLE;
∘ Microaneurysms: saccular aneurysms are dilatation in the top of the loop and the knobs, which are often observed in diabetic microangiopathy;
∘ Neo-angiogenesis: it is a thin, newly formed capillary, which tries to compensate for the disappearance of several loops (Gao et al.).

Abnormalities in the pericapillary space can be:

- Edema, which is detected when it is impossible to focus on the capillaries. When not related to trauma, it indicates severe microangiopathy (6).
- Micro-hemorrhages, which are detected in the pericapillary spaces and progressively move away from the capillary. When they are not of micro-traumatic origin, they indicate an organic microangiopathy. They are frequently found in the vasculitis.
- Exudate: it consists of edema associated with bleeding. It is associated with an organic microangiopathy and predicts a poor prognosis.
- Sweat, which appears as droplets.
- Background color: it is usually pink. It appears pale in Raynaud's anemia and in patients on ß-blocker treatment.
- Sub-papillary venous plexuses are not visible in normal subjects (except pediatric patients). They are visible and prominent in venous hypertension and cor pulmonale and can be seen in acrocyanosis and SLE.

-Flow anomalies include stasis, granular flow, and blood sludging (intravascular clamp of red cells).

-Anomalies in the cold pressure test: this can be the extinction phenomenon observed in Raynaud's phenomenon after the immersion of the hand in cold water (12°C) for 3 min. Arteriolar vasoconstriction occurs, and the capillaries become poorly visible at the NC. It is detected in <10% of normal subjects and 70% of patients with Raynaud's disease (predictive value 95%).

Primary Raynaud's phenomenon is caused by excessive physiological vasospasm due to cold and sympathetic stimuli. There is a difference with the secondary form, which is more often linked to connective tissue diseases. In these cases, the clinical picture is mainly related to the damage to the connective tissue surrounding the capillaries. Therefore, we should speak of a reaction to the cold evocative of Raynaud's phenomenon.

The message is that the NC must be evaluated for the density and alterations of the capillaries, the pericapillary tissue, the characteristics of the fundus, and the flow of the erythrocyte column and the functional tests, which in their entirety can give information about diagnosis and prognosis (Bottino and Bouskela) of cutaneous microangiopathies.

## Author contributions

GT: Conceptualization, Supervision, Writing—original draft, Writing—review and editing. SB: Writing—original draft, Writing—review and editing.
